# Synthesis, X-ray crystal structure, Hirshfeld surface analysis and DFT studies of (*E*)-*N*′-(2-bromo­benzyl­idene)-4-methylbenzohydrazide

**DOI:** 10.1107/S2056989018017978

**Published:** 2019-01-04

**Authors:** Azhagan Ganapathi Anitha, Chidambaram Arunagiri, Annamalai Subashini

**Affiliations:** a PG & Research Department of Physics, Seethalakshmi Ramaswami College, Tiruchirappalli 620 002, Tamil Nadu, India; b PG & Research Department of Physics, Periyar E.V.R. College (Autonomous), Tiruchirappalli 620 023, Tamil Nadu, India; c PG & Research Department of Chemistry, Seethalakshmi Ramaswami College, Tiruchirappalli 620 002, Tamil Nadu, India

**Keywords:** crystal structure, hydrogen bonding, DFT, Hirshfeld surface analysis

## Abstract

The title mol­ecule, C_15_H_13_BrN_2_O, displays a *trans* configuration with respect to the C=N double bond. The dihedral angle between the bromo- and methyl-substituted benzene rings is 16.1 (3)°. In the crystal, mol­ecules are connected by N—H⋯O and weak C—H⋯O hydrogen bonds, forming *R*_2_^1^(6) ring motifs and generating chains along the *a*–axis direction. The optimized structure generated theoretically *via* density functional theory (DFT) using standard B3LYP functional and 6–311 G(d,p) basis-set calculations renders good support to the experimental data. The HOMO–LUMO behaviour was elucidated to determine the energy gap. The inter­molecular inter­actions were qu­anti­fied and analysed using Hirshfeld surface analysis.

## Abstract 2

The title mol­ecule displays a *trans* configuration with respect to the C=N double bond. The dihedral angle between the bromo- and the methyl-substituted benzene rings is 16.1 (3)°. In the crystal, mol­ecules are connected by N—H⋯O and weak C—H⋯O hydrogen bonds, forming 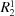
(6) ring motifs and generating chains along the *a*–axis direction.

## Chemical_context   

Hydrazones are a class of organic compounds that possess an *R*1*R*2C=NNH_2_ structural motif. They are related to ketones and aldehydes in which oxygen has been replaced with an NNH_2_ group (Rollas & Küçükgüzel, 2007 ▸). Azomethines, –NHN=CH–, constitute an important class of compounds for new drug development. The reaction of a hydrazine or hydrazide with aldehydes and ketones yields hydrazones. Hydrazones are important in drug design as they act as ligands for metal complexes, organocatalysis and the synthesis of organic compounds. The C=N bond of the hydrazone and the terminal nitro­gen atom containing a lone pair of electron is responsible for the physical and chemical properties. The C atom in the hydrazone unit has both electrophilic and nucleophilic character and both the N atoms are nucleophilic, although the amino-type nitro­gen is more reactive. As a result of these properties, hydrazones are widely used in organic synthesis. Owing to their ease of preparation and diverse pharmacological potential, much work on hydrazones has been carried out by medicinal chemists to develop agents with better activity and low toxicity profiles. Hydrazones are known to possess diverse biological activities such as anti­microbial, anti–inflammatory, anti­cancer and anti­malarial (Yousef *et al.*, 2003 ▸; Trepanier *et al.*, 1966 ▸) and have been evaluated for inhibition of PDE10A, a phospho­diesterase responsible for neurological and psychological disorders such as Parkinson’s, schizophrenia and Huntington’s disease (Gage *et al.*, 2011 ▸). The anti­convulsant potential of some hydrazone derivatives having long duration and rapid onset of action have been reported (Kaushik *et al.*, 2010 ▸), as has their anti-depressant activity (de Oliveira *et al.*, 2011 ▸).

Schiff bases are used widely in the field of coordination chemistry and have inter­esting properties (Morshedi *et al.*, 2009 ▸; Zhou *et al.*, 2006 ▸; Khanmohammadi *et al.*, 2009 ▸). These compounds are synthesized by condensation of carbonyl compounds with amines (van den Ancker *et al.*, 2006 ▸; Hamaker *et al.*, 2010 ▸). In addition, free Schiff base compounds are reported to possess anti­microbial (Aslantas *et al.*, 2009 ▸) and non-linear optical (Karakaş *et al.*, 2008 ▸) properties. Our previous work on (*E*)-4-bromo-*N*′ -(2,4-di­hydroxy­benzyl­idene)benzohydrazide and (*E*)-4-toluic -*N*′-(2,4-di­hydroxy­benzyl­idene)benzohydrazide have been recently reported (Arunagiri *et al.*, 2018*a* ▸,*b* ▸). This work has been a guide for the development of the new Schiff base title compound, which possesses electronic and non-linear properties. As part of our inter­est in the identification of bioactive compounds, we report herein on its crystal structure.
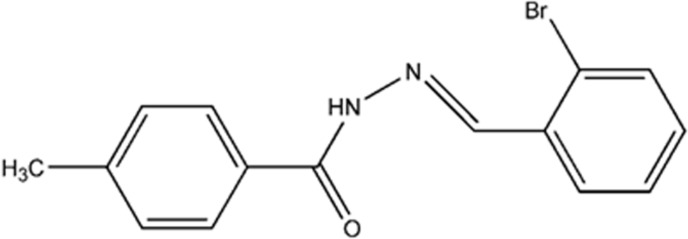


## Structural commentary   

**Figure 1:**
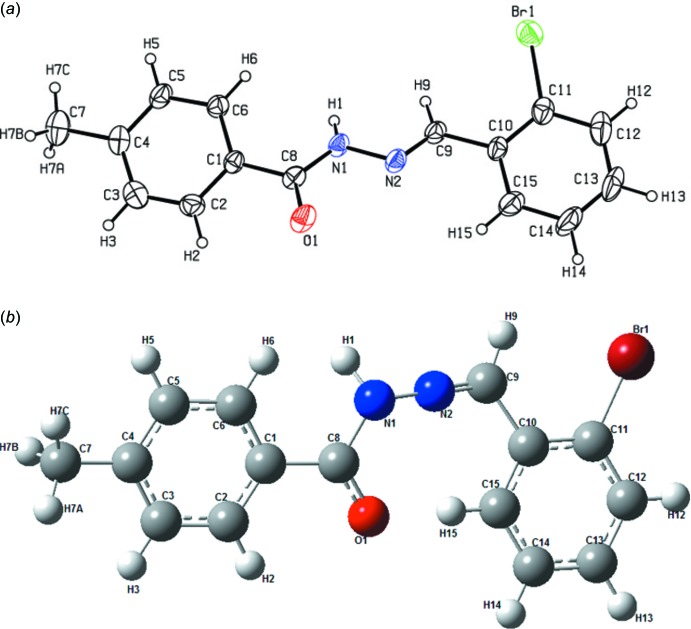
(*a*) The mol­ecular structure of the title compound, with displacement ellipsoids drawn at the 50% probability level. (*b*) The optimized structure of the title compound.

The mol­ecular structure of the title compound is shown in Fig. 1 ▸(*a*). The mol­ecule adopts an (*E*) configuration across the C=N bond, joining the hydrazide group and the benzene ring. In the crystal, the dihedral angle between the bromo- and methyl-substituted benzene rings is 16.1 (3)°. The structure was optimized with the *Gaussian09W* software (Frisch *et al.*, 2009 ▸) using the DFT–B3LYP/6–311G(d,p) method, providing information about the geometry of the mol­ecule. The optimized structure is shown in Fig. 1 ▸(*b*). The geometrical parameters (Table 1 ▸) are mostly within normal ranges, the slight deviations of the theoretical values from those determined experimentally are due to the fact that the optimization is performed in isolated conditions, whereas the crystal environment and hydrogen-bonding inter­actions affect the results of the X-ray structure (Zainuri *et al.*, 2017 ▸).

The hydrazide unit (N1/N2/C1/C8–C10) is essentially planar, with a maximum deviation from the least-squares plane of 0.099 (4) Å for atom C10. The O1=C8 bond length [1.225 (6) and 1.219 Å for XRD and B3LYP, respectively] indicates single-bond character. The N1—N2 bond length [1.379 (5) Å for XRD and 1.364 Å for B3LYP] is in good agreement with other experimental values (Sivajeyanthi *et al.*, 2017 ▸). The C—N bond lengths range from a typical single bond [C8—N1 = 1.351 (6) Å] to a double bond [C9=N2 = 1.270 (6) Å (Sivajeyanthi *et al.*, 2017 ▸; Arunagiri *et al.*, 2018*a* ▸,*b* ▸).

## Supra­molecular features   

**Figure 2:**
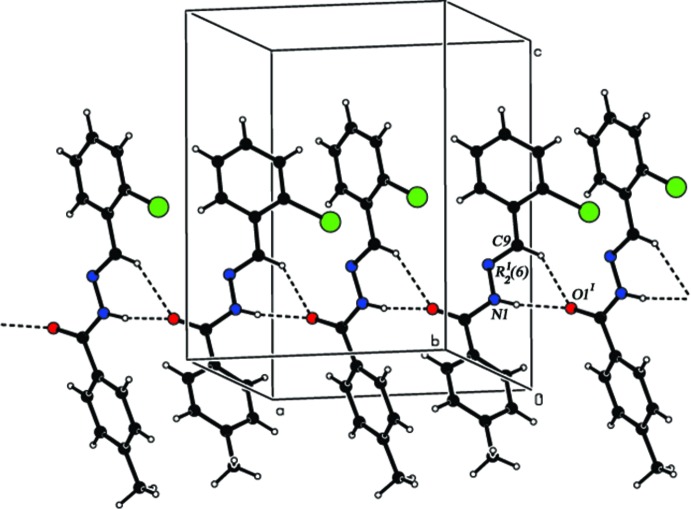
Part of the crystal structure with hydrogen bonds shown as dashed lines.

In the crystal, N1—H1⋯O1^i^ and C9—H9⋯O1^i^ hydrogen bonds (Table 2 ▸) connect symmetry-related mol­ecules through classical N—H⋯O and weak C—H⋯O hydrogen bonds, forming 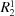
(6) ring motifs and generating [100] chains (Fig. 2 ▸).

## Hirshfeld surface analysis   

**Figure 3:**
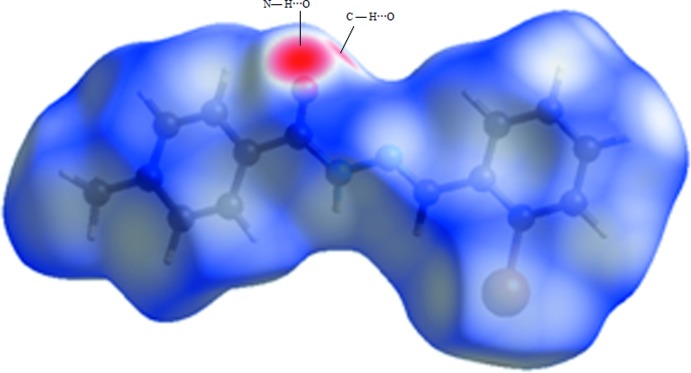
The three-dimensional *d_norm_* surface of the title compound. add contouring levels

**Figure 4:**
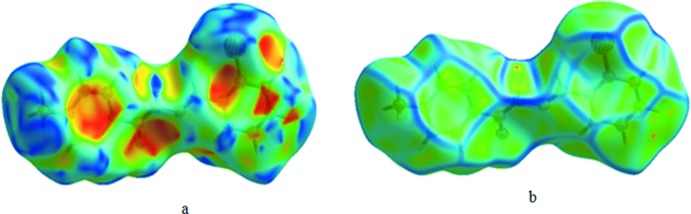
Hirshfeld surfaces mapped over (*a*) shape-index and (*b*) curvedness for the title compound.

**Figure 5:**
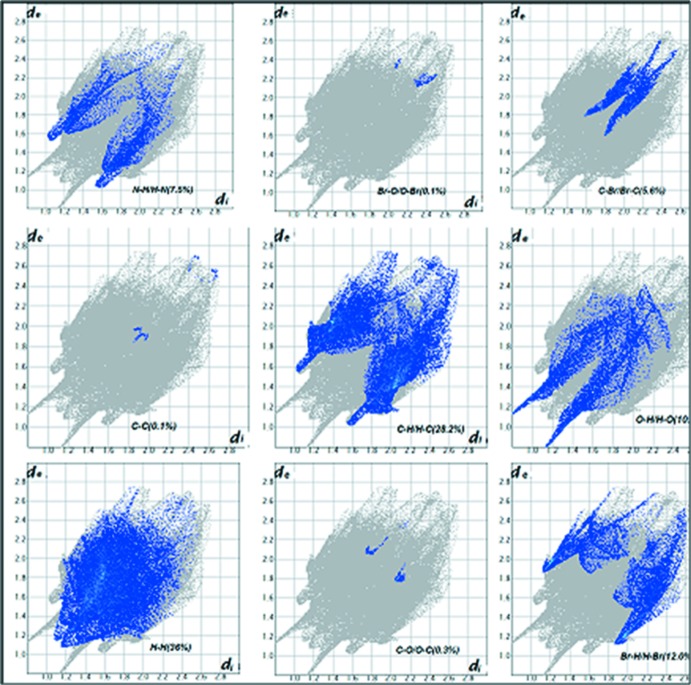
Two-dimensional fingerprint plots with the relative contributions of the various inter­actions.

Hirshfeld surface analysis (McKinnon *et al.*, 2007 ▸; Spackman & Jayatilaka, 2009 ▸) along with decomposed 2D fingerprint plots (Spackman & McKinnon, 2002 ▸; McKinnon *et al.*, 2004 ▸, 2007 ▸) mapped over *d*_norm_, shape-index and curvedness were used to visualize and qu­antify the inter­molecular inter­actions. The Hirshfeld surface (HS) and fingerprint plots were generated based on the *d*_i_ and *d*_e_ distances using *Crystal Explorer3.1* (Wolff *et al.*, 2012 ▸) where *d*_i_ is the distance from the nearest atom inside the surface, while *d*_e_ is the distance from the HS to the nearest atom outside the surface. In the *d*_norm_ surfaces, large circular depressions (deep red) are the indicators of hydrogen-bonding contacts whereas other visible spots are due to H⋯H contacts. The dominant H⋯O inter­action in the title compound is evident as a bright-red area in Fig. 3 ▸ while the light-red spots are due to N—H⋯O and C—H⋯O inter­actions. The shape-index surface [Fig. 4 ▸(*a*)] conveys information about each donor–acceptor pair and while the curvedness surface [Fig. 4 ▸(*b*)] is effectively divided into sets of patches, respectively. The tiny extent of area and light colour on the surface indicates weaker and longer contacts other than hydrogen bonds. The 2D fingerprint plots in Fig. 5 ▸ shows the relative contributions from the various inter­molecular contacts (O⋯H, H⋯H, C⋯H, C⋯C, N⋯H, N⋯N, O.·Br and C.·Br) in the crystal structure. The H⋯H contacts (36%) make the largest contribution, followed by C⋯H/H⋯C (28.2%), O⋯H/H⋯O (10.2%) and N⋯H/H⋯N (7.5%), the latter inter­actions being represented by blue spikes on both sides at the bottom of the plot.

## Frontier mol­ecular orbitals and Mol­ecular electrostatic potential analysis   

**Figure 6:**
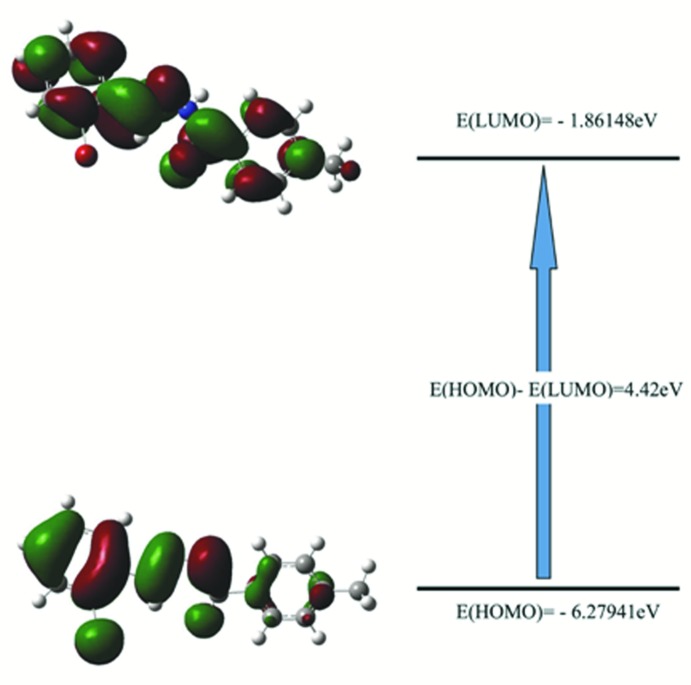
The energy band gap of the compound.

The highest-occupied mol­ecular orbital (HOMO), which acts as an electron donor, and the lowest-unoccupied mol­ecular orbital (LUMO), which acts as an electron acceptor, are very important parameters for quantum chemistry. If the energy gap is small, then the mol­ecule is highly polarizable and has high chemical reactivity. The energy levels were computed by the DFTB3LYP/6-311G(d,p) method (Becke *et al.*, 1993 ▸) as implemented in *GAUSSIAN09W* (Frisch *et al.*, 2009 ▸). The electron transition from the HOMO to the LUMO energy level is shown in Fig. 6 ▸. The mol­ecular orbital of HOMO contain both σ and π electron-density character, whereas the LUMO is mainly composed of π-orbital density. The energy band gap (Δ*E*) of the mol­ecule is about 4.42 eV.

**Figure 7:**
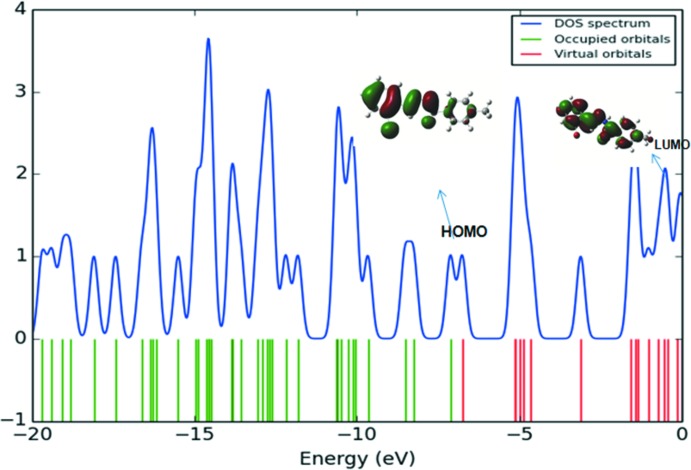
The density of states (DOS) spectrum of the compound.

The *Gauss-Sum2.2* program (O’Boyle *et al.*, 2008 ▸) was used to calculate group contributions to the mol­ecular orbitals (HOMO and LUMO) and prepare the density of states (DOS) spectrum shown in Fig. 7 ▸. The DOS spectrum was formed by convoluting the mol­ecular orbital information with GAUSSIAN curves of unit height. The green and red lines in the DOS spectrum indicate the HOMO and LUMO levels. The DOS spectrum supports the energy gap calculated by HOMO–LUMO analysis. A mol­ecule with a large energy gap is described as hard while one having a small energy gap is known as a soft mol­ecule. Hard mol­ecules are not more polarizable than the soft ones because they require immense excitation energy (Karabacak & Yilan, 2012 ▸).

**Figure 8:**
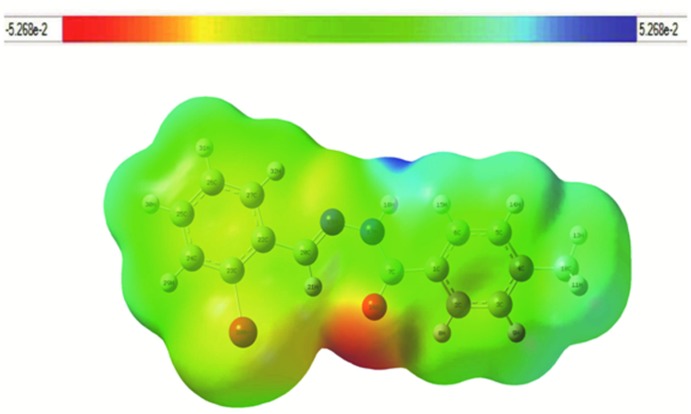
The mol­ecular electrostatic potential map for the title compound.

The mol­ecular electrostatic potential is related to the electron density and mol­ecular electrostatic potential (MESP) maps are very useful descriptors for understanding reactive sites for electrophilic and nucleophilic reactions as well as hydrogen-bonding inter­actions (Sebastian & Sundaraganesan, 2010 ▸; Luque *et al.*, 2000 ▸). Different values of the electrostatic potential are represented by different colours: red represents regions of the most electronegative electrostatic potential, blue represents regions of the most positive electrostatic potential and green represents regions of zero potential. The potential increases in the following order: red < orange < yellow < green < blue. Herein, MEP was calculated at the DFT–B3LYP/6–311(d,p) level of theory that was used for optimization. The MESP map for the title mol­ecule is shown in Fig. 8 ▸ with a colour range from −0.053 (red) to 0.053 a.u. (blue). The most electrostatically positive region (blue colour) is located in the mol­ecular plane (N–bonded hydrogen atoms of toluic hydrazide), thus explaining N1—H1⋯O1^i^ hydrogen bond observed in the crystal structure. The map clearly shows that the electron-rich (red) region is spread around the carbonyl oxygen atom whereas the hydrogen atom attached to nitro­gen is positively charged (blue).

## Database survey   

A search of the Cambridge Structural Database (Version 5.39, last update November 2018; Groom *et al.*, 2016 ▸) revealed closely related compounds that differ in the donor substit­uents: *N*′-(4-chloro­benzyl­idene)-2-hy­droxy­benzohydrazide (Zhang *et al.*, 2009 ▸), (*E*)-*N*′-[(pyridin-2-yl)methyl­ene]benzo­hydrazide (Ramesh Babu *et al.*, 2014 ▸), (*E*)-*N*′-(4-meth­oxy­benzyl­idene)pyridine-3-carbohydrazide dihydrate (Gov­indarasu *et al.*, 2015 ▸), (*E*)-4-bromo-*N*′-(4-meth­oxy­benzyl­idene)benzo­hydrazide(Balasubramani *et al.*, 2018 ▸), (*E*)-3-(1*H*-indol-2-yl)-1-(4-nitro­phen­yl)prop-2-en-1-one hemihydrate (Zaini *et al.*, 2018 ▸); (*E*)-4-bromo-*N*′-(2,4-di­hydroxy­benzyl­idene)benzo­hydrazide and (*E*)-4-toluic-*N*′-(2,4-di­hydroxy­benzyl­idene)benzohydrazide (Arunagiri *et al.*, 2018*a* ▸,*b* ▸). In our studies of analagous hydrazide dervatives (with Cl or Br replacing the methyl group of the title compound, we have observed similar types of Intramolecular *S*(6) and intermolecular N—H⋯O hydrogen bonds (between the amide hydrogen and the carbonyl oxygen atoms).

## Synthesis and crystallization   

**Figure 9:**
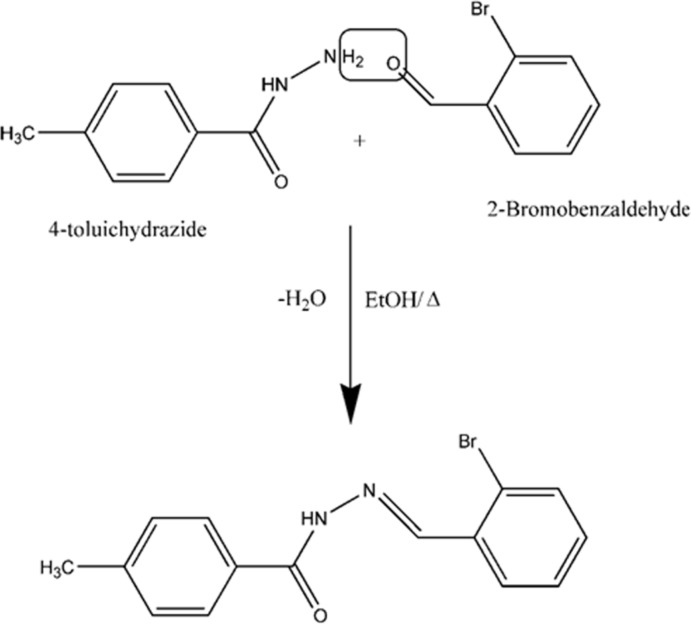
Reaction scheme.

The title compound was synthesized by the condensation of 4-toluic hydrazide and 2-bromo­benzaldehyde (Fig. 9 ▸). An ethanol solution (10ml) of 4-toluic hydrazide (0.25 mol) was mixed with ethanol solution of 2-bromo­benzaldehyde (10 ml, 0.25 mol) and the reaction mixture was heated at 323 K for half an hour with constant stirring before being was filtered and kept for crystallization. After a period of one week, brown block-shaped crystals of the title compound were obtained.

## X-ray crystallography and refinement   

Crystal data, data collection and structure refinement details are summarized in Table 3 ▸. The hydrogen atom on N1 (H1) was located in a difference-Fourier map and freely refined. C-bound hydrogen atoms were placed in calculated positions (C—H = 0.93–0.96 Å) and refined as riding with *U*_iso_(H) =1.2*U*_eq_(C) or 1.5*U*_eq_(C-meth­yl).

## Supplementary Material

Crystal structure: contains datablock(s) global, I, 80R. DOI: 10.1107/S2056989018017978/lh5887sup1.cif

Supporting information file. DOI: 10.1107/S2056989018017978/lh5887Isup2.cml

CCDC reference: 1580647

Additional supporting information:  crystallographic information; 3D view; checkCIF report

## Figures and Tables

**Table 1:** Selected geometric parameters (Å,°) for the experimental and DFT structures

	XRD	DFT
Br1—C11	1.891 (6)	1.925
O1—C8	1.225 (6)	1.219
N1—N2	1.379 (5)	1.364
N1—C8	1.351 (6)	1.383
N2—C9	1.270 (6)	1.285
N1—H1	0.837 (19)	1.006
C1—C8	1.485 (6)	1.501
C9—C10	1.470 (8)	1.470
C9—H9	0.930	1.082
		
N2—N1—C8	119.2 (4)	120.00
C8—N1—H1	123 (4)	117.84
N2—N1—H1	117 (4)	111.31
N1—C8—C1	116.6 (4)	114.46
O1—C8—C1	121.6 (4)	122.20
O1—C8—N1	121.7 (4)	123.33
N2—C9—C10	120.1 (4)	118.62
N2—C9—H9	120.00	122.79
		
C8—N1—N2—C9	−173.3 (5)	−179.68
N2—N1—C8—O1	−3.5 (7)	2.54
N2—N1—C8—C1	174.64)	−178.45
C2—C1—C8—O1	24.6 (7)	22.94
C2—C1—C8—N1	−153.6 (5)	−156.06
N2—C9—C10—C11	161.4 (5)	−179.93
N2—C9—C10—C15	−19.2 (7)	−0.14
C6—C1—C8—N1	26.3 (7)	25.79

**Table 2:** Hydrogen-bond geometry (Å, °)

*D*—H⋯*A*	*D*—H	H⋯*A*	*D*⋯*A*	*D*—H⋯*A*
N1—H1⋯O1^i^	0.84 (3)	1.96 (3)	2.785 (5)	167 (5)
C9—H9⋯O1^i^	0.93	2.38	3.166 (6)	142

Symmetry code: (i) 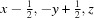.

**Table 3:** Experimental details

Crystal data	
Chemical formula	C_15_H_13_BrN_2_O
*M*_r_	317.18
Crystal system, space group	Orthorhombic, *P* *n* *a*2_1_
Temperature (K)	296
*a*, *b*, *c* (Å)	9.6002 (10), 11.5584 (13), 12.5823 (12)
*V* (Å^3^)	1396.2 (3)
*Z*	4
Radiation type	Mo *K*α
μ (mm^−1^)	2.94
Crystal size (mm)	0.30 × 0.25 × 0.20

Data collection	
Diffractometer	Bruker Kappa APEXII CCD
Absorption correction	Multi-scan (*SADABS*; Bruker, 2004 ▸)
*T*_min_, *T*_max_	0.473, 0.591
No. of measured, independent and observed [*I* > 2σ(*I*)] reflections	17816, 2744, 1837
*R*_int_	0.035
(sin θ/λ)_max_ (Å^−1^)	0.617

Refinement	
*R*[*F* ^2^ > 2σ(*F* ^2^)], *wR*(*F* ^2^), *S*	0.037, 0.097, 1.10
No. of reflections	2744
No. of parameters	176
No. of restraints	2
H-atom treatment	H atoms treated by a mixture of independent and constrained refinement
Δρ_max_, Δρ_min_ (e Å^−3^)	0.42, −0.47

Computer programs: *APEX2*, *SAINT* and *XPREP* (Bruker, 2004 ▸), *SIR97* (Altomare *et al.*, 1999 ▸), *SHELXL014* (Sheldrick, 2015 ▸) and *ORTEP-3 for Windows* (Farrugia, 2012 ▸).

## supplementary crystallographic information

### Crystal data

**Table:**

C_15_H_13_BrN_2_O	*F*(000) = 640
*M**_r_* = 317.18	*D*_x_ = 1.509 Mg m^−^^3^
Orthorhombic, *P**n**a*2_1_	Mo *K*α radiation, λ = 0.71073 Å
Hall symbol: P 2c -2n	Cell parameters from 6594 reflections
*a* = 9.6002 (10) Å	θ = 2.4–26.0°
*b* = 11.5584 (13) Å	µ = 2.94 mm^−^^1^
*c* = 12.5823 (12) Å	*T* = 296 K
*V* = 1396.2 (3) Å^3^	Block, brown
*Z* = 4	0.30 × 0.25 × 0.20 mm

### Data collection

**Table:**

Bruker Kappa APEXII CCD diffractometer	2744 independent reflections
Radiation source: fine-focus sealed tube	1837 reflections with *I* > 2σ(*I*)
Graphite monochromator	*R*_int_ = 0.035
ω and φ scan	θ_max_ = 26.0°, θ_min_ = 2.4°
Absorption correction: multi-scan (SADABS; Bruker, 2004)	*h* = −11→11
*T*_min_ = 0.473, *T*_max_ = 0.591	*k* = −14→14
17816 measured reflections	*l* = −15→15

### Refinement

**Table:**

Refinement on *F*^2^	Primary atom site location: structure-invariant direct methods
Least-squares matrix: full	Secondary atom site location: difference Fourier map
*R*[*F*^2^ > 2σ(*F*^2^)] = 0.037	Hydrogen site location: inferred from neighbouring sites
*wR*(*F*^2^) = 0.097	H atoms treated by a mixture of independent and constrained refinement
*S* = 1.10	*w* = 1/[σ^2^(*F*_o_^2^) + (0.0208*P*)^2^ + 2.2419*P*] where *P* = (*F*_o_^2^ + 2*F*_c_^2^)/3
2744 reflections	(Δ/σ)_max_ < 0.001
176 parameters	Δρ_max_ = 0.42 e Å^−^^3^
2 restraints	Δρ_min_ = −0.47 e Å^−^^3^

### Special details

**Table:**

Geometry. All esds (except the esd in the dihedral angle between two l.s. planes) are estimated using the full covariance matrix. The cell esds are taken into account individually in the estimation of esds in distances, angles and torsion angles; correlations between esds in cell parameters are only used when they are defined by crystal symmetry. An approximate (isotropic) treatment of cell esds is used for estimating esds involving l.s. planes.
Refinement. Refinement of F^2^ against ALL reflections. The weighted R-factor wR and goodness of fit S are based on F^2^, conventional R-factors R are based on F, with F set to zero for negative F^2^. The threshold expression of F^2^ > 2sigma(F^2^) is used only for calculating R-factors(gt) etc. and is not relevant to the choice of reflections for refinement. R-factors based on F^2^ are statistically about twice as large as those based on F, and R- factors based on ALL data will be even larger.

### Fractional atomic coordinates and isotropic or equivalent isotropic displacement parameters (Å^2^)

**Table:**

	*x*	*y*	*z*	*U*_iso_*/*U*_eq_	
Br1	0.27412 (5)	0.49846 (5)	0.50267 (12)	0.06555 (18)	
O1	0.7807 (3)	0.1961 (4)	0.2048 (3)	0.0633 (11)	
N1	0.5643 (4)	0.2641 (4)	0.2337 (3)	0.0430 (10)	
N2	0.5965 (5)	0.2925 (4)	0.3373 (3)	0.0464 (11)	
C1	0.6174 (5)	0.1750 (4)	0.0649 (3)	0.0390 (10)	
C2	0.6886 (5)	0.0846 (4)	0.0191 (5)	0.0529 (14)	
H2	0.7632	0.0514	0.0551	0.064*	
C3	0.6512 (7)	0.0427 (5)	−0.0791 (5)	0.0632 (15)	
H3	0.7000	−0.0193	−0.1080	0.076*	
C4	0.5433 (6)	0.0907 (5)	−0.1352 (4)	0.0546 (14)	
C5	0.4721 (6)	0.1827 (5)	−0.0902 (4)	0.0526 (13)	
H5	0.3987	0.2165	−0.1271	0.063*	
C6	0.5085 (4)	0.2251 (4)	0.0089 (5)	0.0446 (10)	
H6	0.4599	0.2872	0.0378	0.054*	
C7	0.4998 (11)	0.0447 (6)	−0.2424 (6)	0.0834 (19)	
H7C	0.4230	0.0894	−0.2689	0.125*	
H7A	0.4723	−0.0348	−0.2356	0.125*	
H7B	0.5766	0.0503	−0.2910	0.125*	
C8	0.6621 (5)	0.2130 (4)	0.1724 (4)	0.0429 (11)	
C9	0.4964 (5)	0.3296 (4)	0.3937 (4)	0.0446 (12)	
H9	0.4093	0.3413	0.3632	0.053*	
C10	0.5182 (5)	0.3543 (3)	0.5071 (5)	0.0435 (10)	
C11	0.4290 (6)	0.4236 (4)	0.5651 (4)	0.0530 (13)	
C12	0.4514 (8)	0.4419 (5)	0.6726 (4)	0.0726 (19)	
H12	0.3908	0.4882	0.7115	0.087*	
C13	0.5642 (9)	0.3908 (6)	0.7213 (5)	0.082 (2)	
H13	0.5807	0.4039	0.7931	0.098*	
C14	0.6504 (9)	0.3224 (6)	0.6660 (5)	0.083 (2)	
H14	0.7239	0.2857	0.7004	0.100*	
C15	0.6313 (7)	0.3059 (5)	0.5591 (4)	0.0608 (15)	
H15	0.6951	0.2617	0.5210	0.073*	
H1	0.480 (3)	0.268 (5)	0.217 (4)	0.051 (16)*	

### Atomic displacement parameters (Å^2^)

**Table:**

	*U*^11^	*U*^22^	*U*^33^	*U*^12^	*U*^13^	*U*^23^
Br1	0.0673 (3)	0.0624 (3)	0.0669 (3)	0.0056 (3)	0.0118 (6)	−0.0006 (3)
O1	0.0301 (18)	0.111 (3)	0.049 (2)	0.006 (2)	−0.0069 (16)	0.001 (2)
N1	0.033 (2)	0.061 (3)	0.035 (2)	−0.001 (2)	−0.0091 (19)	−0.003 (2)
N2	0.045 (3)	0.058 (3)	0.035 (2)	0.000 (2)	−0.009 (2)	−0.002 (2)
C1	0.035 (2)	0.049 (3)	0.033 (2)	−0.004 (2)	0.001 (2)	0.003 (2)
C2	0.046 (3)	0.058 (3)	0.055 (4)	0.011 (2)	0.003 (3)	0.006 (3)
C3	0.067 (4)	0.059 (3)	0.063 (4)	0.002 (3)	0.008 (3)	−0.008 (3)
C4	0.061 (3)	0.062 (3)	0.041 (3)	−0.013 (3)	0.005 (3)	−0.007 (3)
C5	0.052 (3)	0.066 (3)	0.040 (3)	−0.001 (3)	−0.013 (2)	−0.001 (3)
C6	0.038 (2)	0.055 (3)	0.041 (2)	0.0026 (19)	−0.005 (3)	−0.003 (3)
C7	0.097 (4)	0.095 (5)	0.058 (3)	−0.016 (6)	−0.009 (3)	−0.021 (5)
C8	0.029 (2)	0.059 (3)	0.040 (3)	−0.003 (2)	0.000 (2)	0.008 (2)
C9	0.049 (3)	0.046 (3)	0.039 (3)	−0.003 (2)	−0.013 (2)	−0.002 (2)
C10	0.057 (3)	0.041 (2)	0.033 (2)	−0.008 (2)	−0.006 (3)	−0.002 (3)
C11	0.070 (4)	0.045 (3)	0.043 (3)	−0.014 (3)	0.000 (3)	−0.002 (2)
C12	0.116 (6)	0.059 (4)	0.042 (4)	−0.021 (4)	0.016 (4)	−0.006 (3)
C13	0.152 (7)	0.059 (4)	0.034 (3)	−0.030 (4)	−0.021 (4)	−0.003 (3)
C14	0.117 (6)	0.082 (5)	0.050 (4)	−0.001 (5)	−0.043 (4)	0.002 (4)
C15	0.075 (4)	0.054 (3)	0.053 (3)	−0.002 (3)	−0.020 (3)	0.003 (3)

### Geometric parameters (Å, º)

**Table:**

Br1—C11	1.891 (6)	C6—H6	0.9300
O1—C8	1.225 (6)	C7—H7C	0.9600
N1—C8	1.351 (6)	C7—H7A	0.9600
N1—N2	1.379 (5)	C7—H7B	0.9600
N1—H1	0.837 (19)	C9—C10	1.470 (8)
N2—C9	1.270 (6)	C9—H9	0.9300
C1—C2	1.375 (7)	C10—C11	1.381 (7)
C1—C6	1.389 (6)	C10—C15	1.386 (7)
C1—C8	1.485 (6)	C11—C12	1.385 (7)
C2—C3	1.375 (8)	C12—C13	1.378 (10)
C2—H2	0.9300	C12—H12	0.9300
C3—C4	1.371 (8)	C13—C14	1.340 (10)
C3—H3	0.9300	C13—H13	0.9300
C4—C5	1.386 (7)	C14—C15	1.370 (8)
C4—C7	1.508 (9)	C14—H14	0.9300
C5—C6	1.384 (8)	C15—H15	0.9300
C5—H5	0.9300		
			
C8—N1—N2	119.2 (4)	H7C—C7—H7B	109.5
C8—N1—H1	123 (4)	H7A—C7—H7B	109.5
N2—N1—H1	117 (4)	O1—C8—N1	121.7 (4)
C9—N2—N1	116.0 (4)	O1—C8—C1	121.6 (4)
C2—C1—C6	118.6 (5)	N1—C8—C1	116.6 (4)
C2—C1—C8	117.6 (4)	N2—C9—C10	120.1 (4)
C6—C1—C8	123.8 (5)	N2—C9—H9	120.0
C1—C2—C3	121.0 (5)	C10—C9—H9	120.0
C1—C2—H2	119.5	C11—C10—C15	118.1 (5)
C3—C2—H2	119.5	C11—C10—C9	122.5 (5)
C4—C3—C2	121.2 (5)	C15—C10—C9	119.4 (5)
C4—C3—H3	119.4	C10—C11—C12	120.5 (6)
C2—C3—H3	119.4	C10—C11—Br1	122.2 (4)
C3—C4—C5	118.2 (5)	C12—C11—Br1	117.2 (5)
C3—C4—C7	121.8 (6)	C13—C12—C11	119.4 (6)
C5—C4—C7	120.0 (6)	C13—C12—H12	120.3
C6—C5—C4	121.1 (5)	C11—C12—H12	120.3
C6—C5—H5	119.5	C14—C13—C12	120.5 (6)
C4—C5—H5	119.5	C14—C13—H13	119.8
C5—C6—C1	120.0 (5)	C12—C13—H13	119.8
C5—C6—H6	120.0	C13—C14—C15	120.6 (7)
C1—C6—H6	120.0	C13—C14—H14	119.7
C4—C7—H7C	109.5	C15—C14—H14	119.7
C4—C7—H7A	109.5	C14—C15—C10	120.8 (6)
H7C—C7—H7A	109.5	C14—C15—H15	119.6
C4—C7—H7B	109.5	C10—C15—H15	119.6
			
C8—N1—N2—C9	−173.3 (5)	C6—C1—C8—N1	26.3 (7)
C6—C1—C2—C3	−1.5 (7)	N1—N2—C9—C10	175.7 (4)
C8—C1—C2—C3	178.4 (5)	N2—C9—C10—C11	161.4 (5)
C1—C2—C3—C4	1.0 (8)	N2—C9—C10—C15	−19.2 (7)
C2—C3—C4—C5	−0.2 (8)	C15—C10—C11—C12	−1.2 (7)
C2—C3—C4—C7	−179.2 (6)	C9—C10—C11—C12	178.2 (5)
C3—C4—C5—C6	−0.1 (8)	C15—C10—C11—Br1	178.1 (4)
C7—C4—C5—C6	178.9 (6)	C9—C10—C11—Br1	−2.5 (6)
C4—C5—C6—C1	−0.3 (8)	C10—C11—C12—C13	0.4 (8)
C2—C1—C6—C5	1.1 (7)	Br1—C11—C12—C13	−178.9 (5)
C8—C1—C6—C5	−178.8 (5)	C11—C12—C13—C14	−1.1 (10)
N2—N1—C8—O1	−3.5 (7)	C12—C13—C14—C15	2.8 (11)
N2—N1—C8—C1	174.6 (4)	C13—C14—C15—C10	−3.6 (10)
C2—C1—C8—O1	24.6 (7)	C11—C10—C15—C14	2.8 (8)
C6—C1—C8—O1	−155.5 (5)	C9—C10—C15—C14	−176.6 (6)
C2—C1—C8—N1	−153.6 (5)		

### Hydrogen-bond geometry (Å, º)

**Table:**

*D*—H···*A*	*D*—H	H···*A*	*D*···*A*	*D*—H···*A*
N1—H1···O1^i^	0.84 (3)	1.96 (3)	2.785 (5)	167 (5)
C9—H9···O1^i^	0.93	2.38	3.166 (6)	142

Symmetry code: (i) *x*−1/2, −*y*+1/2, *z*.
